# Digitalization, stress, and social worker–client relationships during the COVID-19 pandemic

**DOI:** 10.1177/14680173231180309

**Published:** 2023-06-15

**Authors:** Kettil Nordesjö, Gabriella Scaramuzzino

**Affiliations:** Malmö University, Malmö, Sweden; Lund University, Lund, Sweden

**Keywords:** Social work, stress, video-conferencing, social workers, mixed methods, work

## Abstract

**Summary:**

The COVID-19 pandemic has dramatically changed the possibilities for people to interact and communicate. This article examines Swedish social workers’ experiences of the extent to which the COVID-19 pandemic has affected the use of digital tools in their work, and whether this use has affected the social worker–client relationship and their stress levels. The article draws on a web survey (N  =  541) via a quantitative analysis of responses and a qualitative analysis of answers to an open-ended question.

**Findings:**

Most respondents agreed on experiencing increased use of digital tools in the relationship with the clients, increased skills in using digital tools, and a more positive view of digital tools in the social worker–client relationship. However, experiences on whether stress levels had increased and the relationship with the clients worsened, were divided. Age correlates positively with increased stress levels, and social workers working with social assistance, as well as women, are more likely to agree on that the relationship with the clients has worsened. Responses from open-ended questions highlight a rapid shift where social workers have gained a more positive view of digital tools, that video meetings can increase efficiency and flexibility, but also work environment problems.

**Applications:**

This article contributes with useful insights into how the use of digital tools during the COVID-19 pandemic has changed and affected stress and the social worker–client relationship. It can support discussions on the future implementation of digital tools in social work after the pandemic.

## Introduction

Like most countries, there is strong political pressure in Sweden to increase and accelerate the pace at which welfare services are digitalized. There has been a concern that the development is not going fast enough, as it has proven difficult to implement new technology, especially within social services (Scaramuzzino & Hjärpe, 2021; Scaramuzzino, 2022). A survey study from the beginning of 2020, right before the onset of the COVID-19 pandemic, shows that a majority of the municipalities in Sweden had developed digitalization strategies and specially-assigned staff. Of these, 40% of municipalities used e-applications for social assistance support, and 2% used them for child welfare. Only a few (12% and 9%, respectively) offered secure digital communication channels to their clients, and even fewer had chat forums where citizens could ask social workers anonymous questions (National Board of Social Affairs and Health, 2020, p. 7, 15–16). The COVID-19 pandemic dramatically changed the need for people to interact and communicate, which is why it is important to further explore to what extent social workers had to change their ways of working to meet the needs of clients, but also to what extent the pandemic affected their work environment.

It is reasonable to assume that this rapid transformation may challenge elements of the social worker–client relationship such as face-to-face interaction. This relationship has traditionally been understood as an integral part of social workers’ professional identity and purpose (Rollins, 2020) and has been described as the very core of an intervention, or as a service in and of itself (Gray et al., 2012). Social workers’ well-being is essential for building and maintaining these relationships, and poor working conditions and high stress levels can have a negative impact on social workers’ attitudes toward clients, and hence the social worker–client relationship (see Blomberg et al., 2015; Scaramuzzino & Martinell Barfoed, 2021).

Research on the significance of social workers’ use of digital tools for the social worker–client relationship is growing (see Nordesjö et al., 2022 for an overview). Here, digital tools used in the social worker–client relationship refer to tools that support, govern, or replace social work practice in case management, outcome measurement, interventions, and communication (see Mackrill & Ebsen, 2018). There are also several studies on the transformative social implications of the COVID-19 pandemic for different areas of social work (see e.g., Cheung, 2022; Sullivan-Tibbs et al*.*, 2022, for systematic reviews and Dallas Allen et al., 2021; Ross et al., 2021 for special issues), but the knowledge on how the COVID-19 pandemic has contributed to digitalization's significance for the social worker–client relationship is inconclusive. While some research concludes that social workers have been able to maintain a therapeutic relationship with clients (Mishna et al., 2020), others claim that the quality of client care has been reduced by the use of telehealth services (Wiener et al., 2021). Also, clients with complex problems—like mental health problems and loneliness—were the most negatively affected by the COVID-19 pandemic (Mesiäislehto et al., 2021; see also Ross et al., 2021). Also, there are few studies exploring how various factors (e.g., gender, age, work tasks, employment, and size of the municipality) may explain the impact on the social worker–client relationship, which is something that this article contributes. Since the goal of digital interventions is often “maintaining or promoting health, wellbeing, quality of life and/or increasing efficiency in the operational delivery of welfare, social and health care services, while improving working conditions of the staff” (Richardson et al., 2022, p. 1), this article will focus both on the social worker–client relationship and on social workers’ stress levels. As has already been discussed, social workers’ stress levels can impact the relationship and the outcome of digital interventions.

The aim of this article is to examine social workers’ experiences of the extent to which the COVID-19 pandemic has affected the use of digital tools in their work, and whether this use has affected the social worker–client relationship and their stress levels. Drawing on a web survey distributed to Swedish social workers (n  =  541), we answer the following research questions:

1. To what extent has the COVID-19 pandemic affected social workers’ use of digital tools in their work, the social worker–client relationship, and their stress levels?
2. What factors (gender, age, work tasks, employment, and size of the municipality) explain whether social workers experience that the use of digital tools during the COVID-19 pandemic has affected the social worker–client relationship and their stress levels?
3. How do social workers experience that the COVID-19 pandemic has affected their use of digital tools, the social worker–client relationship, and their stress levels?

## The social worker–client relationship in the digital and COVID-19 era

### Digitalization's significance for the social worker–client relationship

Research on digitalization's significance for the social worker–client relationship can be grouped into three perspectives (Nordesjö et al., 2022). Studies coming from an *interpersonal* perspective are characterized by understanding relationships as psychosocial interactions and communication between people, often referring to relationship-based, therapeutic, or psychodynamic social work (e.g., Lopez, 2015; Simpson, 2017; Turner, 2016). A *contextual* perspective sees digital tools as external policies or interventions that may threaten relational and narrative social work within an administrative, organizational, or political context. Social workers can use their discretion to deal with rules and regulations and develop strategies to achieve a responsive social work practice (e.g., Breit et al., 2021; Devlieghere & Roose, 2018; Devlieghere et al., 2020). This perspective is more concerned with understanding how various preconditions for the social worker–client relationship are shaped. Also, a *critical* perspective is employed in studies exploring digital tools as informal surveillance techniques, and how technology can transform clients’ everyday life and its subsequent ethical challenges (e.g., Lim, 2017; Mortenson et al., 2015). In this article, we adopt a pluralistic view of the social worker–client relationship. Research within an interpersonal and contextual perspective can shed light on different aspects of the relationship without contradicting each other. Also, we did not define the social worker–client relationship in the web survey, which means that the respondents may have had different understandings of its meaning.

There is little research explaining differences in social workers’ experiences of digitalization's significance for the social worker–client relationship. One reason may be that social workers’ use of technology is related to the organization in which they work, rather than to the individual (Goldkind et al., 2016), or that much research is qualitative, where background variables such as *gender* are of descriptive rather than explanatory interest (e.g., Harris, 2022). Few studies have highlighted social workers’ *age*. One example is Mishna et al. (2021) who found age and years of practice to be significantly related to informal Information and Communication Technologies (ICT) use with clients in Canada and the U.S. Also, a study on American child welfare found younger social workers to be more confident when using mobile technologies, although they were less enthusiastic about the role that technology would play in client engagement (Whitaker et al., 2010). Finally, research on digitalization's significance for the social worker–client relationship rarely compares social workers’ *work tasks* (see Mishna et al., 2021 for an exception).

### Digitalization's significance for the social worker–client relationship: Stress

On the other hand, there is extensive research on social workers’ experiences of job stress (see e.g., Blomberg et al., 2015; Hussein, 2018). As working life has become more digitalized, researchers have also started to focus on “technostress” in social work, even if it is difficult to distinguish between “general stress,” “job stress,” and “technostress” (see e.g., Ragu-Nathan et al., 2008; Scaramuzzino & Martinell Barfoed, 2021). “Technostress” refers to stress caused by different types of technology; for example, a full inbox, or computer lags (Scaramuzzino & Martinell Barfoed, 2021). There are also studies on “digital coping”; that is, how frontline workers cope with encounters that are digitally mediated (e.g., Breit et al., 2021). Much focus has been on the use of Electronic Information Systems and how they can lead to stress but also limit the relational aspects of social work (e.g., Devlieghere & Roose, 2018). However, current research on social workers’ experiences of stress at work shows diverse results, and it has been discussed how digital tools such as ICTs can both create and ease job stress levels (see Scaramuzzino & Hjärpe, 2021 for an overview).

A survey study on Swedish social workers’ experiences of technostress discusses how gender, age, and education influence experiences of technostress. In particular, contextual factors related to work tasks and working conditions explained whether they experienced technostress. The findings show that one-third of social workers experienced technostress, especially when the boundaries between work and non-work domains were blurred (Scaramuzzino & Martinell Barfoed, 2021; see also Breit et al., 2021). The use of certain digital tools such as smartphones and laptops enable agile work and the flexibility to work in different places. Although agile work can create a sense of isolation and an increase in work pace and stress levels, it is also associated with freedom, convenience, and pleasure (Jeyasingham, 2019). Another study that compares social workers working in Children and Adult Services, found that gender and ethnicity are associated with burnout, and that work engagement and perceived level of resources are important factors for feelings about your work, regardless of your main target group (Hussein, 2018). It has also been discussed how the COVID-19 pandemic, and the fact that people were advised to work from home and have digital meetings, affected stress levels (Scaramuzzino & Martinell Barfoed, 2021), but this has not yet been empirically investigated.

### Digitalization's significance for the social worker-client relationship: COVID-19

The COVID-19 pandemic left millions of service users isolated when typical social work interventions could not be performed as intended (Cheung, 2022). Scholars have discussed how communication with clients through digital tools rapidly increased (see e.g., Dallas Allen et al., 2021; Ross et al., 2021). Social work interventions have been reconfigured to be delivered online, such as telehealth services that facilitated and increased access to services (Senreich et al., 2021). Research differs as to whether this shift has reduced or increased the quality of services. Mishna et al. (2020) discuss how digital tools have provided flexibility for professionals and clients, and thus helped maintain a therapeutic relationship (see also Cook et al., 2020; Swenson et al., 2021). However, Wiener et al. (2021) found that social workers believed that telehealth services reduced the quality of client care. For example, Cook and Zschomler (2020) found that virtual home visits in child welfare cases were perceived to have significant limitations for initial assessments. Also, a decrease in face-to-face work hinders professionals’ ability to identify and respond to issues of concern (Driscoll et al., 2020).

Moreover, professional boundaries have been challenged due to the increased use of digital tools (Mishna et al., 2020; Senreich et al., 2021; Sullivan-Tibbs et al., 2022). Social workers have been forced to rethink how their professional values can be applied in new contexts (Cheung, 2022) and how working remotely can ensure social workers’ “availability, sensitivity, acceptance, cooperation and a sense of belonging” (Cook et al., 2020, p. 260; see also Morse & Dell, 2021). Consequently, the need for social work training and education in the use of digital tools has been raised (Swenson et al., 2021; Wang et al., 2021), and social workers should be able to tailor digital services to specific needs.

In sum, digitalization's significance for the social worker–client relationship during the COVID-19 pandemic is inconclusive. Also, there is little to no research explaining differences in social workers’ experiences of digitalization's significance for the social worker–client relationship. There is extensive research on social workers’ experiences of job stress, and the number of studies on technostress is growing. However, the COVID-19 pandemic created a situation where the use of digital tools became crucial, which is why it's important to investigate to what extent this new situation affected stress levels.

## Methods and data: A web survey among Swedish social workers

This article is based on a survey study that was conducted between April 25 and May 26, 2021. A web survey was sent out to a sample of 5,000 members of the Union for Professionals. At the time of the study, the Union for Professionals had more than 72,000 members and organized academics in social sciences and was the largest union for social workers in Sweden (see Union for Professionals, 2021). Only professional social workers were included in the sample, and they received an email with a link to the web survey, followed by two reminders. See Table 1.

**Table 1.** Sample size and response rate.

	Social workers
Sample (emails sent out)	5,000
Respondents	541
Response rate	10.82%

In the Swedish context, the response rate is low. Previous survey studies on Swedish social workers have had a response rate of 60% in 2008 (Meeuwisse et al., 2011) and 22% in 2018 (Scaramuzzino & Martinell Barfoed, 2021), but there is a current development of shrinking response rates when it comes survey studies. To test the representativeness, a dropout analysis of respondents was conducted by comparing the respondents in the web survey to the Union's members, based on members’ statistics in terms of gender, age, education, and employment sector. See Table 2.

**Table 2.** Dropout analysis of the respondents.

	Population (n =** ** 5,000)	Respondents (n =** ** 541)
Percentage of women	89%	89%
Age 20–29	16.9%	12.9%
Age 30–39	41.0%	32.0%
Age 40–49	22.4%	24.8%
Age 50–59	14.2%	21.3%
Age 60−	5.4%	9.0%
Age, average	39.6	42.7
Percentage with a social worker degree	88%	90%
Percentage employed in the public sector	99%	100%
Percentage in municipalities > 200k	26%	17%

As Table 2 shows, there is no bias in terms of gender distribution and no major bias when it comes to either social worker degree or employment sector distribution. However, when it comes to age distribution among the members of the Union for Professionals and respondents, there is a bias. Social workers who are over 50 years old are over-represented, while social workers under the age of 39 are under-represented in the web survey. Fewer respondents also work within the smaller municipalities. Based on this dropout analysis, even if there are some biases in the composition of the respondent group, we argue that these few differences do not have any major impact on the representativeness of the data. As age is used as a factor in the analysis, we will address possible consequences of age differences between the population and respondents for the results in the concluding part.

### Measures and data analysis

The analysis is divided into two parts: a survey question and free-text responses. In the first part, the following question was asked: *Below there are statements about the significance of COVID-19 on your work. Choose the alternative that best suits you. Answer based on the digital tools that you use. The COVID-19 pandemic has contributed to*… The question had six statements (see Table 3) with the following response alternatives: (1) strongly agree; (2) somewhat agree; (3) neither agree nor disagree; (4) somewhat disagree; (5) strongly disagree. The term ‘digital tools’ was used as a broad concept to include all types of digital tools. We specified that the focus was on digital tools used in the relationship with the client (except for the statement on stress, which was also general). The free-text responses have been used to nuance the broad concept of “digital tools” by highlighting what types of digital tools the respondents referred to.

**Table 3.** Actual situation and effects %.

	Strongly agree	Somewhat agree	Neither agree or disagree	Somewhat disagree	Strongly disagree	Total N.
… I more often use digital tools in the relationship with the client	71.1	19.9	3.8	2.3	3.0	532
… I gained more competence about how digital tools can be used in the relationship with the client	32.3	41	15.4	4.5	6.8	532
… I have become more positive about digital tools in the relationship with the client	24.7	42.6	20.7	7.7	4.3	531
… more digital tools for use in the relationship with the client have been implemented at my working place	34.5	37.7	13.4	5.1	9.2	530
… my relationship with clients has worsened	11.5	35.8	27.7	12.6	12.3	530
… my stress from digital tools has increased	8.3	23.3	24.7	15.7	28.1	530

As a first step, a descriptive analysis was conducted to analyze the respondent's experience of how the COVID-19 pandemic had changed their actual situation and its effects (RQ1). As a second step, a binary logistic regression analysis was conducted to test then-correlations between the COVID-19 pandemic and factual (e.g., I more often use digital tools) and perceived factors (e.g., my relationship with clients has worsened) (RQ2). This type of analysis required that these response alternatives were dichotomized, which was done by merging some of the alternatives as positive answers (strongly agree and somewhat agree) and negative answers (neither agree nor disagree, somewhat disagree, and strongly disagree). By analyzing the data in this way, it was not possible to measure to what extent the respondents agreed or disagreed with the statements made in the questionnaire, only to measure whether they agreed. Five of the statements focused on the social worker–client relationship, while one statement was about stress levels. The results regarding the statements “my stress from digital tools has increased” and “my relationship with clients has worsened” stood out in the descriptive analysis and have been used as dependent variables in the binary logistic regression analysis. Drawing on previous research on both the social worker–client relationship and technostress, a few independent variables were chosen, focusing on both the respondent's background (gender and age) and work (work task, size of the municipality, and full time/part time).

The second part of the analysis is based on N  =  333 free-text responses (61.6% of the respondents in the web survey) to the question: *How would you say your conception of the use of digital tools in your work has changed due to the COVID-19 situation*? Some responses were just a few words, such as “more positive” but most were one sentence or more. Responses were coded into themes. The coding process was inductive, occurring by grouping and regrouping the responses according to the subject to which they referred. However, the final five themes were ultimately closely related to the six statements (one theme related to two statements), presumably because the respondents chose to nuance their responses to the statements rather than to discuss additional subjects. However, we also made a second analysis of the free-text responses to understand why some groups stood out in the quantitative analysis. The comments made by male, female, younger, and older social workers, as well as social workers working in social assistance and child welfare, were therefore compared. This analysis is important because it can help to explain some of the results of the survey question.

## Results

### Actual situation and effects

Most of the respondents agreed that the COVID-19 pandemic had changed their actual situation, but they clearly had different opinions on the effects of the COVID-19 pandemic. See Table 3.

As Table 3 shows, 90% of the social workers responded that they agree or partly agree that they use digital tools more often in their relationships with clients due to the COVID-19 pandemic. Almost three out of four participants responded that more digital tools that can be used in the relationship with clients have been implemented due to the COVID-19 pandemic. The results suggest that there has been an increase in the use of digital tools in the social worker–client relationship. Also, about three in four social workers responded that they had developed greater competence regarding how digital tools can be used in the social worker–client relationship, and two in three social workers responded that they have been more positive about using digital tools in the social worker–client relationship. Therefore, the results suggest increased skills in using digital tools and a more positive view of using digital tools in the social worker–client relationship. However, as shown in the lower part of Table 3, opinions on what the effects have been are more divided. Almost half of the respondents answered that they agree or partly agree that the use of digital tools during the COVID-19 pandemic has contributed to worsening relationships with clients. Almost one in three respondents answered that their stress levels had increased due to the COVID-19 pandemic.

As discussed above, we have chosen to use “increased stress levels” and “worsening relationship with clients” as dependent variables. First, we will investigate to what extent the two dependent variables are correlated to each other. A bivariate analysis using Pearson's correlation coefficient shows a strong correlation (0.364**). It's not possible to say anything about causation, but there is a connection, and theoretically, we can understand that there is an impact in both directions. We, therefore, tested both these dependent variables against different independent variables to be able to examine which factors seem to be important for each dependent variable. The following independent variables were included in the regressions: gender, age, work task (social assistance, addiction, and childcare), size of the municipality, and full-time/part time employment. See Table 4.

**Table 4.** Correlations between the COVID-19 pandemic and factual and perceived factors.

	Increased stress levels (strongly agree, somewhat agree)		Worsening relationship with clients (strongly agree, somewhat agree)	
	Standard error	Exp(*B*) (odds ratio)	Standard Error	Exp(*B*) (odds ratio)
*Background variables*				
Gender (female ref.)	0.320	0.551^†^	0.318	0.493*
Age 30–39 (20–29 ref.)	0.410	2.464*	0.312	1.222
Age 40–49 (20–29 ref.)	0.417	3.252*	0.323	1.366
Age 50–59 (20–29 ref.)	0.424	3.584**	0.332	1.393
Age 60 + (20–29 ref.)	0.482	5.992***	0.412	1.614
*Work-related variables*				
Social assistance/financial support (no use ref.)	0.313	1.666	0.282	1.954*
Substance abuse/addiction (no use ref.)	0.312	0.619	0.268	0.828
Child welfare and family support (no use ref.)	0.290	1.198	0.259	0.844
Employment (full time ref.)	0.273	1.192	0.262	1.457
Municipality 3-4 = 1 ()	0.222	1.339	0.206	1.125
Municipality 5 = 1 ()	0.303	1.093	0.278	1.111
Constant	0.557	0.195**	0.482	1.081
Observations	485	485		
Nagelkerke *R*^2^	0.086	0.066		

*Note*. † = 10%, * = 5%, ** = 1%, *** = 0.1%.

There is a clear correlation between increased stress levels and age. The older the group, compared to the youngest, the higher the odds that they would agree to that they have experienced increased stress levels from using digital tools during the COVID-19 pandemic. Men are less likely than women to experience that their relationship with clients has worsened. Social workers working in social assistance are more likely to agree on that their relationships with clients have worsened than are social workers working in other tasks or areas.

Some of these results are *not* consistent with previous research. A survey study on Swedish social workers’ experiences of technostress (before the COVID-19 pandemic) shows that a majority of social workers experienced different degrees of technostress, but there was almost no correlation regarding either age or gender. Social workers who experienced general job stress, an excessive workload or that the boundaries between work and non-work domains were blurred, were more likely to experience technostress (Scaramuzzino & Martinell Barfoed, 2021). More general studies on technostress show that factors such as age, gender, and computer confidence matter, and that older people usually experience lower technostress levels than younger people that have been explained by their increased ability to cope with stress and change than their younger colleagues, given their more extensive life experience (e.g., Ragu-Nathan et al., 2008). No previous quantitative research is available to understand the correlations with the second dependent variable—whether the relationship has worsened.

### How the COVID-19 pandemic affected the use of digital tools and the social worker–client relationship

The free-text responses were much in line with the results of the survey question, but add important nuances.

#### A rapid shift: Implementation and use of new digital tools

The first theme relates to whether more digital tools for use in the relationship with the client have been implemented due to the COVID-19 pandemic. The respondents describe how the COVID-19 pandemic and demands on social distancing led to a rapid change in the ways in which social workers worked, communicated, and interacted with their clients:

A dramatic increase! From having only physical meetings and certain phone calls/text messages between meetings to having investigations via video meetings and phone calls as standard and almost no physical meetings. (R434)

Several respondents also describe how the COVID-19 pandemic accelerated the digital transformation of social work in a way they previously thought impossible: “Before the pandemic, there was no question about working from home and booking digital meetings in social services” (R432). The digital tools most-often referred to are video meetings and video conferencing: “According to me, the use of digital tools has increased enormously, and has become ‘normal’ in many workplaces” (R227). A few social workers explain that they haven’t experienced a major change at the workplace, but overall, the comments on this theme are much in line with research on the COVID-19 pandemic and social work in other countries that experienced this rapid shift (e.g., Cheung, 2022; Mishna et al., 2022).

#### The more social workers use digital tools, the more positive they become about using them

The second theme relates to the finding regarding that the more social workers have used digital tools, the more positive they have become about using them: “I’ve learned that digital meetings often work just as well or even better than physical meetings” (R82). Digital tools such as digital meetings contribute to less travel, increased time efficiency, and new possibilities of making contact with clients. Such contributions resonate with the findings of Mishna et al. (2022), who found that the use of ICT provided practitioners with the flexibility to meet and handle client meetings, making it possible to maintain the social worker–client relationship. Also, several respondents argue that prioritizing has become easier: “For the better. I scrutinize myself more regarding which meetings are actually necessary to have” (R53). Others explain that they used to be ignorant and uncomfortable, but have changed their viewpoint due to more experience, skills, and knowledge:

I’m basically an old lady with a negative attitude, but some things actually get better. When I have physical meetings with clients, I’ve been able to ‘connect’ partners from other authorities, which is a big advantage. Also, with COVID-19, you don’t have to cancel meetings and can meet with some participants digitally. Unfortunately, Teams doesn’t meet the standard for security for ensuring confidentiality, so we have another called Compodium. It works like crap and isn’t user friendly. Lacks features like raising the hand, etc. If I’d been able to use Teams and Zoom, I’d have been more positive. (R139)

Using user-friendly digital tools may, therefore, make social workers more positive. Certainly, research suggests that the adaptation of ICT to social work practice is facilitated by specifying purposes, possibilities, and the content of electronic communication between the social worker and the client so that social workers have a clear idea of how to use ICT (Recmanová & Vávrová, 2018). Still, a few social workers emphasized that they had gained a more negative view of digital tools or that their view is unchanged, while others emphasized both positive and negative aspects:

[I] see both advantages and disadvantages of using digital tools in social work. I think the most optimal is a combination of digital tools, but also enabling physical meetings if the client wishes it. (R502)

The quote draws attention to how different digital tools can be beneficial to the social worker–client relationship in different contexts or under different conditions.

#### Physical versus digital meetings

The third theme relates both to how digital tools have been used more often in the relationship with the client, and how the relationship with clients may have worsened because of it.

Respondents reflected on the differences between physical and digital meetings, and in line with previous research, their views were divided (e.g., Price-Robertson et al., 2019; van de Luitgaarden & van der Tier, 2018). Face-to-face meetings were often described as essential for developing a meaningful relationship. Digital meetings are “used more often at the expense of physical encounters” (R57) and physical encounters are preferable: “It's easier to make a better assessment of the right to assistance when having physical meetings with clients. It's easier to assess a person's ability or lack of ability at physical meetings” (R142).

Digital meetings could have contributed to worsening the social worker–client relationship. Certain groups have been difficult to meet since digital tools are “not adapted for clients with addiction problems and the difficulties that this group has” (R447). In particular, social workers working in social assistance, who more often agreed that the social worker–client relationship has worsened due to changes necessitated by the COVID-19 pandemic, often described how it was difficult to meet certain clients and client groups in digital meetings. One explanation is that management had not provided adequate technology, resulting in interrupted meetings. It could also be argued that professionals in social assistance meet clients with a wide range of social problems that may suffer from standardized, straightforward, and brief interactions more often inherent in digital meetings (see van de Luitgaarden & van der Tier, 2018). There are therefore different reasons as to why some respondents think social worker–client relationships have worsened during the COVID-19 pandemic.

Other respondents describe the advantages of digital meetings: “The possibility of getting in contact has improved even during the pandemic. Those who are afraid still have the opportunity to ‘meet’ via the computer” (R423). Also, digital meetings “enables a contact where dialogue can take place and see each other through video, which contributes to a feeling of greater closeness” (R242). Such experiences resonate with research on *social presence*, theorizing how relationships can be maintained through technology by feeling connected (e.g., Simpson, 2017). Offering alternative ways of communication can ideally contribute to clients’ sense of control and empowerment (e.g., Bolin & Sorbring, 2017; Denby et al., 2016). In this sense, digital meetings can be a *complement* to physical meetings:

I think it's good that we can offer more alternatives for our clients in addition to meet in person. The younger generation is already used to using digital tools and does not think it's at all strange to use Skype, for example. However, I still think that the physical meeting is most important, if possible. (R213)

This reasoning is in line with earlier research that emphasizes the flexibility of ICT in social work (e.g., Cwikel & Friedmann, 2020; Mishna et al., 2020). Several social workers emphasized how digital communication contributes to both increased *efficiency* and *flexibility,* which were described as positive for the social worker–client relationship: “It's easier to meet, both for me and the client, fewer cancellations” (R71). Also, digital communication enables meetings with more people, sometimes at the same time. Saving time by not having to travel was described as rewarding for the social workers, but also for the clients: “More digital meetings, which means that the client can avoid travel time and therefore also becomes more accessible as they can share, for example, during a break at work” (R103).

#### Agile working, technical problems, and increased stress levels

The fourth theme relates to a statement in the survey focusing on increased stress levels. Several of the respondents describe how they were advised to work from home after the COVID-19 pandemic outbreak. In contrast to extensive research on boundaryless work (see e.g., Scaramuzzino & Martinell Barfoed, 2021), most of the comments were positive about more agile working practices in social work (see also Jeyasingham, 2019): “I have been able to continue working because I belong to a risk group, so I’ve been allowed to stay at home full time” (R280). Some respondents describe how what was impossible before is suddenly possible: “In the past, according to EVERYONE, it was technically impossible to work remotely. When the pandemic came, it took three days to solve this technical impossibility” (R474). Others described how working from home had made their work more “structured”:

More work from home, I meet the working group more often, as we meet every day. It feels like the working group has become closer to each other. Peace and quiet to work and stay focused without being interrupted when working from home. (R322)

However, there are also downsides in working from home. Only having digital meetings can make the work ‘boring’, and some feel trapped: “Clients use email to communicate with me to a greater extent. This has both pros and cons, but it ties up my time more at the computer…” (R274). In line with much research on technostress (see e.g., Scaramuzzino & Martinell Barfoed, 2021), several digital meetings in a row are exhausting:

Less time spent on, for example, collaboration meetings, but fewer micro-breaks in everyday life; you book one meeting after another. More time in front of the screen  =  more headaches and fatigue after work. (R346)

Working from home with your children was described as especially stressful:

I have been able to work when the children have been home with mild symptoms. But it's not good in the long run. It has been stressful to manage the children's schooling in parallel with my own remote work. (R163)

Some social workers also describe technical problems and lack of support: “Increased frustration due to how dependent I am on technology and the employer refuses to provide useful tools while increasing demands on documentation and availability” (R248). Also, problems with privacy issues and guaranteeing confidentiality are described:

How do we ensure that the child is alone? It's the same with victims of violence—challenges in ensuring that they are protected and safe during the conversation. Very challenging and many conversations about how we can work are conducted. (R533)

Overall, these problems may be rooted in digital tools setting the term for social work, rather than the other way around, stressing the need for social workers to play a more active role in participatory design processes (Mackrill & Ebsen, 2018). Left unsolved, these problems situations can cause “ethical stress” (see e.g., Scaramuzzino & Hjärpe, 2021). Older social workers, who had described experiencing increased stress levels more often during the COVID-19 pandemic in the web survey, noted how they did have access to digital tools, but that they were difficult to use without education and pre-existing competence in using them.

#### Digital competence

The last theme relates to a statement in the questionnaire about to what extent the social workers had experienced that they had gained more competence (e.g., knowledge and skills) about how digital tools can be used in the relationship with the client; that is, digital competence: few responses can be related to this statement. Consistent with previous research, they emphasize the role of digital skills in the relationship between social workers and clients (Lavié & Fernandez, 2018; López Peláez et al., 2020), but also in relation to stress levels. A respondent wrote: “We had to develop new methods and ways of working and the digital tools have helped us. Have accelerated to a positive development that facilitates” (R404). What the comments show here that is not present in previous research, is the discussion that employers must also improve and increase their digital competence: “Employers need more knowledge on how the digital tools that we use affect the work environment. The employees need more digital skills. This competence varies greatly among employees with the same position” (R328).

## Limitations of the study

There are limitations to the study. The response rate is low, and the study only focuses on Swedish social workers’ experiences. Also, digital tools within social work refer to different technologies which are used for different purposes and work tasks. In the questionnaire, we did not distinguish between different types of digital tools. In five of the six statements, we specify that the focus is on digital tools used in the relationship with the client. It is important to stress that digital tools that are primarily used to interact with clients and digital tools that are used for administrative tasks could potentially give different results. Nevertheless, by including the free-text responses in the analysis, we have been able to both nuance and specify what type of digital tool respondents were referring to.

## Discussion

This article contributes knowledge on some of the implications that changes in technology, accelerated by the COVID-19 pandemic, have had for social work practice in Sweden. Findings related to our first research question show that the COVID-19 pandemic has affected social workers’ use of digital tools in their work, the social worker–client relationship, and their stress levels. Social workers claim that more digital tools have been implemented and used more often, to have gained greater digital skills, and that they, in general, have become more positive about using digital tools. However, whether their relationship with clients has worsened and whether their stress levels have increased, is debatable and leads us to the second research question regarding factors which explain social workers’ experiences. In particular, women and social workers in social assistance more often claim to perceive worsened relationships with their clients, and older social workers experienced increased stress. As the age factor turned out to be relevant for explaining stress levels, and the dropout analysis of the respondents shows a small bias concerning age, it is reasonable to assume that the results about stress levels for the respondents are slightly higher than in the general population. Qualitative data related to our third research question regarding how social workers experience that the COVID-19 pandemic has affected their use of digital tools, the social worker–client relationship, and their stress levels, have nuanced the study's other findings. Findings show a rapid shift in the implementation of digital tools and how social workers have become more positive about the use of tools, since they can provide flexibility and efficiency. Social work organizations and social workers quickly adapted to the new conditions and implemented new digital tools ‘overnight’. Still, findings show increased stress levels and technical problems related to the use of digital tools, which resonate with research on the difficulties of implementing digital tools within social work, especially within the social services (Scaramuzzino & Hjärpe, 2021; Scaramuzzino, 2022; National Board of Social Affairs and Health, 2020).

The results in this article can be understood from different research perspectives on digitalization's significance for the social worker–client relationship. In an *interpersonal* perspective—characterized by understanding relations as psychosocial interactions and communication between people—the results describe how the COVID-19 pandemic has increased social workers’ use of digital tools, and they have ultimately come to appreciate them. New digital tools complement face-to-face meetings and improve the social worker–client relationship through increased accessibility and flexibility for both professionals and clients. Problems such as stress and the worsening of relationships could ideally be prevented through social work training and workplace discussions on ethics.

In a *contextual* perspective—where digital tools are seen as external policies challenging relational social work within its administrative, organizational, or political context—problems of stress and worsening social worker–client relationships can be understood through insufficient technological, professional, and legal support structures. Failure to provide sufficient technology and low digital competence will hinder the implementation of digital tools in the relationship. An unreflective and simplified implementation of digital tools may push values of efficiency and accountability (e.g., more frequent and less expensive digital meetings) that may not benefit the social worker–client relationship. Instead, future implementation could rely on collaborative design processes clarifying the purposes, possibilities, and content of digital tools between the social worker and the client.

Finally, in a *critical* perspective, digital tools may contribute to a potential increase in multi­directional surveillance of clients, but also of social workers. For example, ambitions regarding client empowerment may also be vehicles involving monitoring and increased administration, obscuring and further tipping the power balance in favor of social workers. Although ambitions regarding empowerment are present in our data, reflections on monitoring and surveillance are not.

Hence, there are different ways of understanding the findings that can help in discussing what lessons can be learned from the COVID-19 pandemic in terms of how digital tools can be used in the social worker–client relationship in the future. Will almost two years’ worth of experience where digital meetings were the ‘norm’ have a long-term influence and long-term effects on both the view of the social worker–client relationship and how social work should be performed, or was it just a parenthesis before going back to “business as usual”? In line with previous research on the COVID-19 pandemic and social work (see also Mesiäislehto et al., 2021; Ross et al., 2021), the social workers in the web survey stated that digital communication makes the services more accessible for some target groups and clients, while for other target groups and clients, it's just the opposite. However, clients’ experiences of how they benefit from or are hindered by the increased use of digital tools requires more research. Similarly, comparative research could investigate the contexts or conditions where different digital tools have beneficial effects for the social worker–client relationship (Nordesjö et al., 2022).
